# Multiple helical configuration and quantity threshold of graphene nanoribbons inside a single-walled carbon nanotube

**DOI:** 10.1038/srep13741

**Published:** 2015-09-16

**Authors:** Yifan Li, Wei Chen, Hongru Ren, Xuyan Zhou, Hui Li

**Affiliations:** 1 Key Laboratory for Liquid-Solid Structural Evolution and Processing of Materials, Ministry of Education, Shandong University, Jinan 250061, People’s Republic of China

## Abstract

Molecular dynamics simulation has been carried out to explore the configuration and quantity threshold of multiple graphene nanoribbons (GNRs) in single-walled carbon nanotube (SWCNT). The simulation results showed that several GNRs tangled together to form a perfect spiral structure to maximize the π-π stacking area when filling inside SWCNT. The formation of multiple helical configuration is influenced by the combined effect of structure stability, initial arrangement and tube space, meanwhile its forming time is related to helical angle. The simulated threshold of GNRs in SWCNT decreases with GNR width but increases with SWCNT diameter, and two formulas have come up in this study to estimate the quantity threshold for GNRs. It has been found that multilayered graphite is hard to be stripped in SWCNT because the special helical configuration with incompletely separated GNRs is metastable. This work provides a possibility to control the configuration of GNR@SWCNT.

Over the past decade, considerable efforts have been devoted to exploring various heterostructures with a combination of SWCNT and its fillers because some modifications to the physical property are expected from their interaction. The hollow interior of SWCNT^1^ can serve as nanometer-sized molds or templates^2^,^3^ in material fabrication or as a protective layer^4^ to prevent the filler from oxidation and shape fragmentation. Filling SWCNTs with chosen fillers can produce one-dimensional nanostructures with exciting new applications^5^ because the quasi-one-dimensional nanoscale structures confined in a nanotube have novel properties quite different from those of bulk materials^6^,^7^. A giant amount of studies have been performed to investigate the encapsulation of some fillers into SWCNT, such as various metal atoms^8^,^9^, halides^10^, C_60_^11^, carbon nanoring^12^, polyacetylene^13^ and La_2_@C_80_ composite^14^, etc. Importantly, incorporation of water into SWCNTs has been demonstrated both theoretically^15^,^16^,^17^ and experimentally^18^ under ambient conditions. Koga *et al.*^19^ found that, when confined inside a SWCNT under axial pressure, water can exhibit a first-order freezing transition to hexagonal and heptagonal ice nanotubes which shows spiral configuration, same as DNA^20^,^21^ inside SWCNT.

Recently, both theoretical^22^,^23^ and experimental^24^ results also demonstrated that GNR, the material with unique magnetic, optical and electrical properties^25^,^26^,^27^,^28^, has been succeed in spirally inserting into the SWCNT. Further simulations^29^ revealed that the typical helical configuration of GNRs is available if the diameter of SWCNT was larger than the sum of the width of GNR and twice the length of a C-H bond. The integration of GNR@SWCNT composite structure has aroused more and more interests because it was expected to exhibit some specific and predominant properties for applications in biochemical and nano-electronical realms^30^,^31^. For example, Irina *et al.*^32^ took advantage of the sulfur-terminated graphene nanoribbon to produce nanowires protected by nanotube wall or inductance nanocoils because the deformation of the sulfur-terminated zigzag GNR inside SWCNTs was insufficient to open the band gap. In addition, an interesting experiment^33^ was reported to fabricate the GNR@SWCNT composite by polycyclic aromatic hydrocarbon molecules using confined polymerization inside nanotubes. And new carbon nanotubes could be synthesized by spiral GNRs through bonding their dehydrogenated edge^34^, providing a method to prepare multi-walled carbon nanotubes and generate H_2_. In the process of new material synthesis in the SWCNT cavity, it is crucial to know that how many fillers can be stored in SWCNT and how to achieve that. Till now, although there were much progress in the understanding of the GNR@SWCNT evolution, some important questions, such as the quantity threshold and special configuration of multiple GNRs encapsulated in SWCNT as well as their influence factors, have remained unanswered. In this work, multiple GNRs have been filled into SWCNT to study their self-assembly behavior, which provides an opportunity for a comprehensive and satisfactory understanding of how to control the composite structure of this SWCNT-based nanomaterial hybrid to produce nanoscale mass transport.

## Results

**Figure 1:**
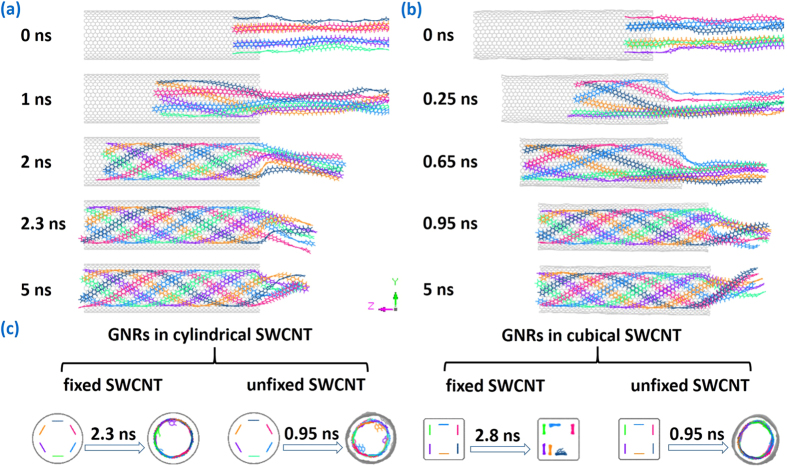
The insertion and helix-forming process of six GNRs into SWCNT: (**a**) snapshots of GNRs encapsulated in the fixed SWCNT; (**b**) snapshots of GNRs encapsulated in the unfixed SWCNT; (**c**) effect of the SWCNT configurations on the evolution process.

The evolution process of multiple GNRs encapsulated into SWCNT as well as their final structures is illustrated in Fig. 1. The GNR with the opening edge is prepared by cutting the parallel sheet of block graphite, and its size is 5.681 Å × 147.600 Å. The length of SWCNTs is 98.38 Å. Direct simulations in Fig. 1(a) show the self-assembly of six GNRs into the fixed SWCNT. At the beginning, As six GNR heads fill into the cavity of SWCNT synchronously and spontaneously under the action of van der Wall force, the tails of GNRs are affected by each other to collapse as a result of the attractive, noncovalent π-π interaction^35^ between their six-membered rings. When the simulation time is up to 2.3 ns, a clear and symmetrical multiple helical configuration with uniform pitch is formed in SWCNT, like an upgrade of the double helix of DNA^36^ or the triple helix of collagen^37^ in biology, suggesting nature’s preference for helical structure. Meanwhile the essential corrugation of GNRs^38^ is suppressed by the enhanced interaction between GNRs and SWCNT. Then as the encapsulating and tightly packing of GNRs are terminated, no more axial movement has been further observed for the helical GNRs due to the van der Waals potential well, but rotation is continued in their circumferential direction, causing the residual GNRs outside SWCNT to keep a new subtle spiral morphology by movement inertia. The filling snapshots of six GNRs into the unfixed SWCNT is established in Fig. 1(b). With the multiple GNRs and the unfixed SWCNT moving oppositely under the shared van der Waals force, a slight deformation of carbon nanotube appears followed by the formation of a perfect single helix of GNRs inside the unfixed SWCNT at 0.95 ns. It takes shorter time for the unfixed SWCNT-GNR system to attain equilibrium, illustrating that the deformation and movement of carbon nanotubes do not hinder the formation of helical structure, but improve the filling speed of GNRs.

**Figure 2:**
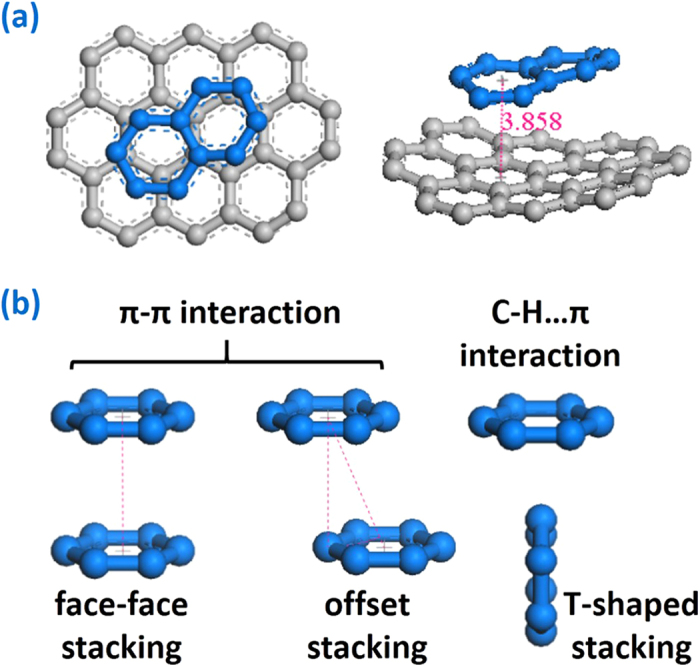
(**a**) Partial enlarged detail of the GNR@SWCNT multiple helical configuration where SWCNT is grey and GNR is blue; (**b**) three representative conformations of the π–π stacking.

In the crowded environment, long molecular chains frequently adopt ordered, helical conformations. Snir and Kamien^39^ provide that the enlarged environment entropy drives helix formation to reduce total energy for a solid, impenetrable tube in a solution of hard spheres. However, as for the GNRs inside SWCNT, spontaneous filling and intertangling are driven by the increasing π-π stacking area between GNRs and SWCNT because system energy is released when carbon six-membered rings stacking. To confirm this view, a series of simulation results acquired from six GNRs encapsulating different SWCNTs are shown in Fig. 1(c). Similar multiple helical GNRs are taken in cylindrical SWCNT shell to maximize π-π stacking area, meanwhile the special cubical fixed SWCNT obtains the flat GNRs which can guarantee the largest stacking area with straight stationary tube wall. The partial enlarged detail of the GNR@SWCNT helical configuration in Fig. 2(a) demonstrates that the GNR ring plane is slightly offset from SWCNTs’ and their centroid–centroid distance is 3.858 Å, according with the typical aromatic–aromatic offset π-π stacking^40^ which is lower in energy than other stacking arrangements. The three representative conformations of six-membered rings stacking are shown in Fig. 2(b).

**Figure 3:**
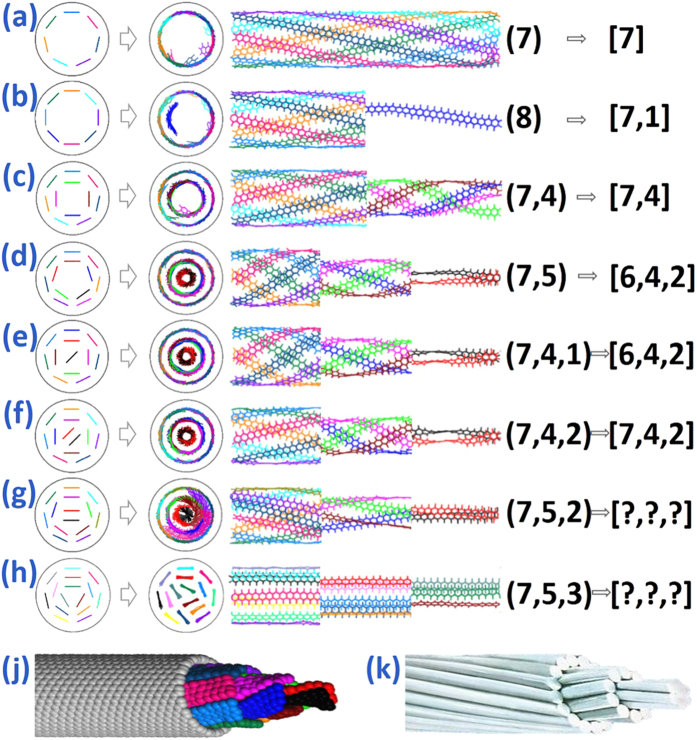
The initial arrangement and equilibrium configuration of the annular and multilayer GNRs in SWCNT. From (**a**) to (**h**), the number of GNRs is 7, 8, 11, 12, 12, 13, 14 and 15 respectively; (**j**) Corey-Pauling-Koltun configuration of the [7, 4, 2] GNRs; (**k**) configuration of the stranded conductor in cable.

To further research the special configuration and quantity threshold (maximum supported number of spiraling) of the multiple GNRs encapsulated in SWCNT, different numbers of annular and multilayer GNRs filling into SWCNT are shown in the Fig. 3. For convenience, we set a new and canonical definition for (a_1_, a_2_…a_i_…a_N_) and [a_1_, a_2_…a_i_…a_N_] where a_i_ means the number of GNRs in i-layer in SWCNT while parenthesis and square brackets refer to the simulated model and final result respectively. 7 GNRs form a monolayer helical configuration in SWCNT in Fig. 3(a), while Fig. 3(b) shows that there is a GNR deviating from the first helix into the second one instead of forming the perfect single spiral structure with other seven GNRs, suggesting that the simulated threshold of the first helix is 7. Meanwhile the twisted angle of inner GNRs is easily affected by the outer one to ensure the maximum overlap area between GNRs in different helices when only one GNR in the inner helix, as the royal blue GNR in Fig. 3(b). But when several GNRs in the inner helix, their twisted angle does not get affected. This phenomenon might help one adjust or control the spiral morphology of GNRs in SWCNT. Similar method is also used to judge the threshold value of the GNRs in the second helix as 4 by comparing Fig. 3(c)–(e). When encapsulating the (7, 4, 2) GNRs into SWCNT in Fig. 3(f), multiple helical GNRs arrange densely with same space (about 3.5 Å) to obtain a perfect multislice spiral structure, and all of the GNRs in the outer layer screw in the outer helix while the inner one screws in the inner helix. When setting more GNRs in SWCNT like Fig. 3(g), the filling process is also spontaneous but the helical configuration becomes imperfect due to their undistinguished layer. In addition, the well-organized multislice spiral structure of [7, 4, 2] consists of an array of such helicoids formed concentrically (shown in Fig. 3(j)), quite similar to the configuration of stranded wire in cable (shown in Fig. 3(k)), so the GNR@SWCNT composite structure could be expected to have excellent performance in mechanics and electrics like stranded wire.

**Figure 4:**
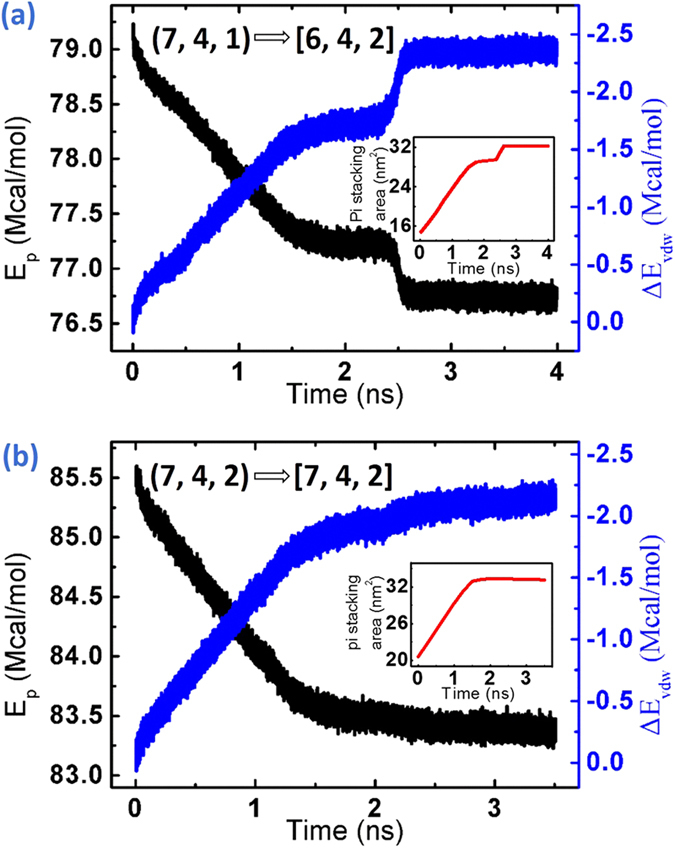
The potential energy (E_p_) of GNR@SWCNT system and the van der Waals interaction energy (E_vdw_) between the GNR and SWCNT as functions of simulation time in the process of helical encapsulation, and the inserted graph is the corresponding evolution of π-π stacking area.

**Figure 5:**
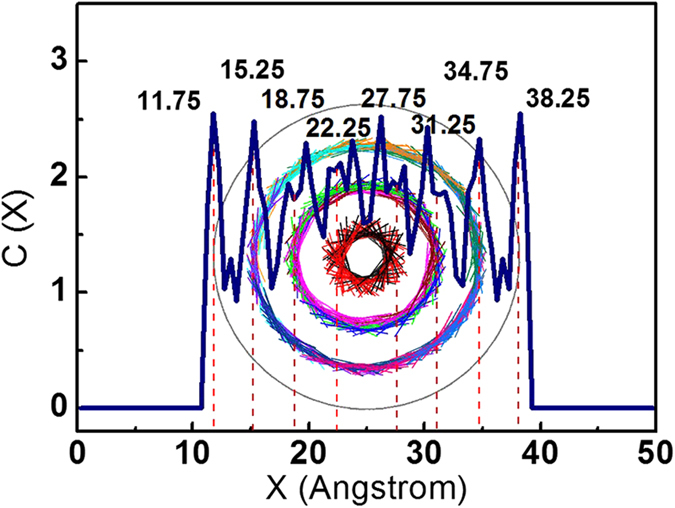
Concentration distribution profiles of the GNR and SWCNT in the [7, 4, 2] system in the X direction.

To further explore the energy change in the encapsulating process, the potential energy (E_p_), van der Waals interaction energy (E_vdw_) and π-π stacking area for the GNR@SWCNT system are shown in Fig. 4. The evolution of π-π stacking area and van der Waals energy are identical, demonstrating that the energy decreasing is caused by the π-π stacking area expanding. The decline of potential energy illustrates that the inserting and spiraling process of the GNRs are spontaneous which makes the system more stable. As for the (7, 4, 1) GNRs in Fig. 4(a), both two energy curves are divided into two stages which correspond to two structural changes. In the first stage from 0 ns to 1.8 ns, multiple GNRs fill into carbon nanotube synchronously along their original directions. Then two GNRs transfer into inner helical structure individually from the first and the second helix in the second stage from 2.3 ns to 2.6 ns, so that the configuration of system is changed from the [7, 4, 1] to [6, 4, 2] with a rapid decrease of potential energy, indicating that the process of GNRs changing into inner helix can improve their stability when space is sufficient. In this process, the van der Waals energy drops 0.7 Mcal/mol while the total potential energy drops just 0.5 Mcal/mol, indicating that the excess energy is stored efficiently by the bending of new inner GNRs. A clear change of energy gradient is shown in Fig. 4(b) about the (7, 4, 2) GNRs into SWCNT. The initial large energy gradient is contributed by rapidly expanding π-π stacking areas as multiple GNRs filling until 1.3 ns when GNRs reach the another end of the carbon nanotube, then the energy gradient decreases because tails of GNRs slowly insert the SWCNT space which is vacated by GNRs spiraling and configuration optimizing. In addition, the concentration distribution profiles in Fig. 5 are created to help collect the parameters of the multiple helical configuration. Although the peak of concentration distribution profiles is splitted near the center of carbon nanotube owing to the uneven edges and the tilt of the carbon atoms in GNRs caused by the rigidity of C-C bond and the larger curvature in the inner helix, it can be measured roughly that the distance between SWCNT and GNRs or neighboring GNRs in different helix is 3.5 Å, similar to the basal plane separation in graphite.

**Figure 6:**
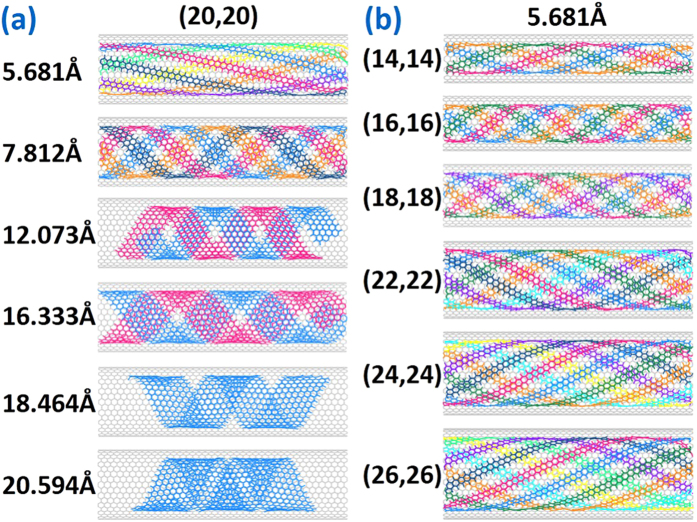
The dependence of the diameter of SWCNT and the width of GNRs in the evolution: (**a**) helical encapsulation of GNRs with different width into the SWCNT (20, 20); (**b**) helical encapsulation of GNR into the SWCNT with different diameter.

**Figure 7:**
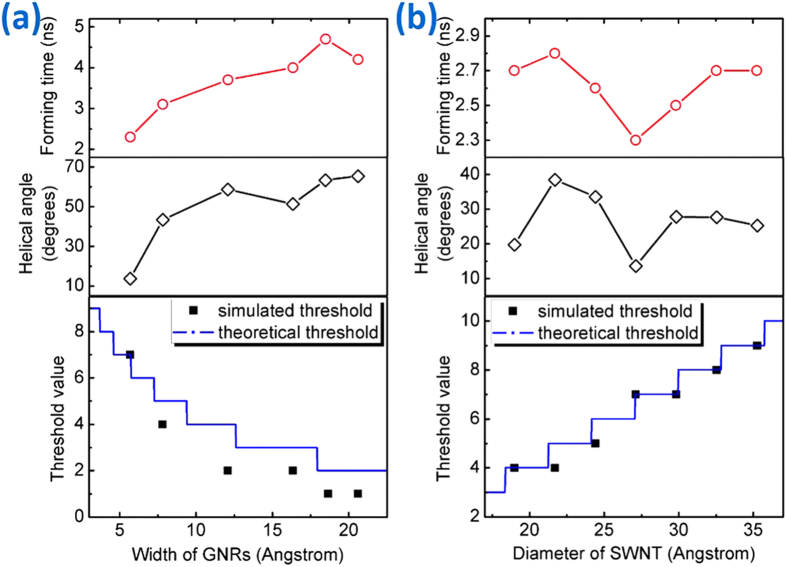
(**a**) Relation between the width of GNRs and the forming time, helical angle or threshold value of the GNR@SWCNT monolayer helical configuration; (**b**) relation between the diameter of SWCNT and the forming time, helical angle or threshold value of the GNR@SWCNT monolayer helical configuration.

In order to further study the size influence of SWCNT and GNRs on helix formation and filling threshold, a series of simulations and parameter analyses have been completed as shown in Fig. 6 and Fig. 7. All monolayer helical configurations have been guaranteed to correspond to their simulated threshold because some of GNRs in the first layer will enter into inner if increasing the number of GNRs. It can be seen from Fig. 6(a) that all GNRs with different widths in the SWCNT of different diameters could form the perfect helical structure, while the GNR pitch in different systems is varying. Configuration forming time and GNR helical angle increase with GNR width, but have no clear monotone change tendency with SWCNT diameter, as shown in Fig. 7. And the curve of forming time is similar to the curve of helical angle, illustrating that more time is needed for the formation of a large helical angle in consideration of multiple GNRs motion and coordination. Besides, relation between the size of SWCNT or GNRs and threshold value is also shown in Fig. 7. The simulated threshold of GNRs in the first perfect helix is inverse with their width owing to the limited space inside SWCNT, ranging from 7 for very narrow width GNRs to 1 for the widest available GNRs in SWCNT (20, 20). In Fig. 7(a), by comparison between the simulated threshold (black spots) with the theoretical maximum supported number (blue line) whose corresponding calculation method is explained in the next discussion part, it is found that some former values are smaller than the latter, which are induced by the stochastic motion of wide GNRs outside of the SWCNT to decrease the system energy in enough interspace. Meanwhile Fig. 7(b) shows that the increase of the simulated threshold value (black spots) is generally accompanied by an increase in diameter of the carbon nanotube, which is nearly consistent with the theoretical one (blue line) when the width of GNRs is 5.681 Å since it is harder to be inducted by their narrow tails to enter the inner helix than the wide one.

**Figure 8:**
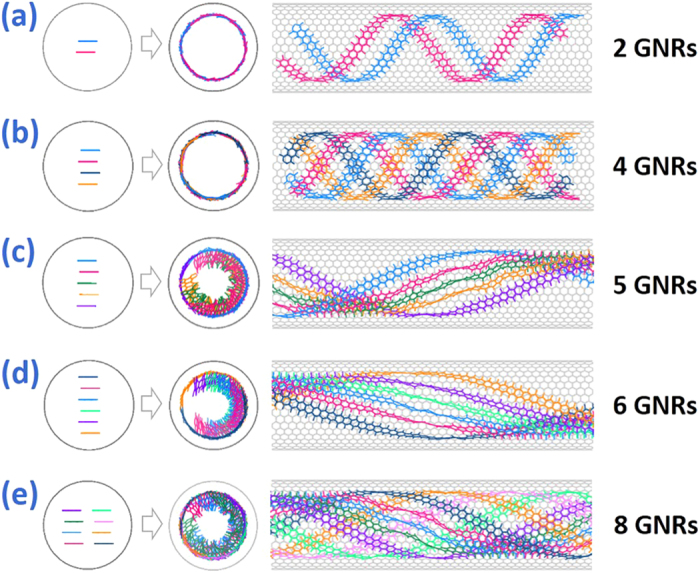
The initial arrangement and equilibrium configuration of the multilayered graphite with different number of GNRs in SWCNT. From (**a**) to (**h**), the number of GNRs is 2, 4, 5, 6 and 8 respectively.

To figure out whether the multilayered graphite has been stripped by the π-π stacking interaction in SWCNT, another simulation has been performed. Figure 8(a) show that two or four layered GNRs are separated from each other to close to the inner wall of SWCNT and further form a perfect single helix structure, suggesting that the interaction between GNRs and SWCNT is much stronger than the one between adjacent GNRs. However 5 and 6 GNRs also hold a helix tendency but not disperse to develop a perfect spiral structure. Similarly, if 8 GNRs with partial separation filling SWCNT, there is a helical hybrid where two kinds of GNRs are parallel or perpendicular to the inner wall of carbon nanotube in the first layer of spiral structure, as shown in Fig. 8(e). The main limitation of the multiple layered GNR separation is insufficient energy fluctuation in system when there are large number of GNRs, because the helical hybrid configuration could be considered as a metastable state which is hard to decompose because there are not only the π-π stacking attractive interaction between GNR layers, but also the enthalpically favorable T-shaped π interactions^40^ between SWCNT and perpendicular GNRs. The hybrid-filler composite structure may have different properties and applications from perfect multiple configuration.

## Discussion

**Figure 9:**
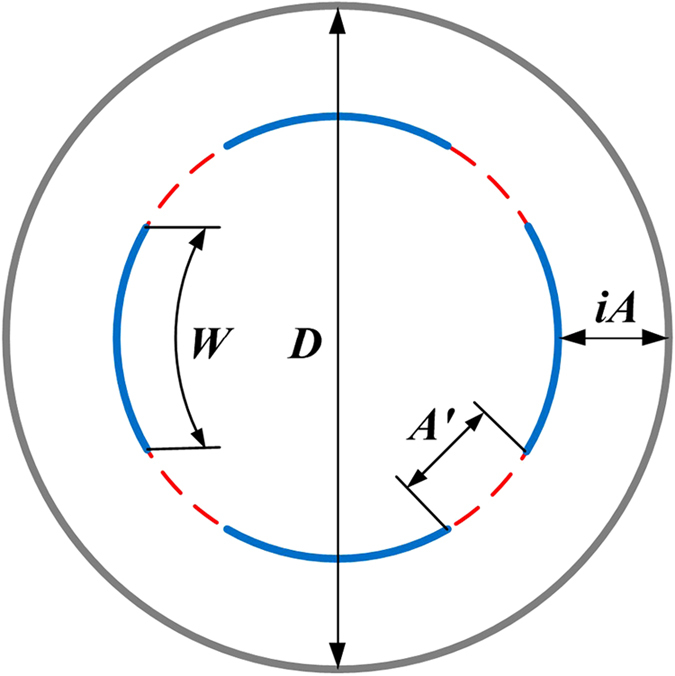
The ideal model of the cross section of GNR@SWCNT helical structure. SWCNT and GNR are referred to as grey circle and blue arc respectively.

As the concentration distribution profiles in Fig. 5 indicate that the distance between SWCNT and GNRs or neighboring GNRs in different helix is 3.5 Å, a simple mathematical model could be built to calculate the quantity threshold for the GNRs encapsulated in SWCNT. Analysis using the ideal model of GNR@SWCNT in Fig. 9 gives two formulas as follows.

The threshold value of GNRs in *i*-layer is


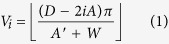


Where *D* is the diameter of carbon nanotubes; *i* is the sequence of the helix of GNRs; *A* is the fixed distance between GNRs and SWCNT; *A’* is the fixed distance between adjacent turns of GNRs; *W* is the width of GNRs, and the specific square brackets means that the calculated value is rounded down to the nearest whole unit.

The whole threshold value of GNRs in SWCNT is


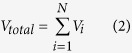


Where *N* is the number of helix of GNRs in SWCNT, which is described by


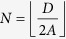


Formulas 1 and 2 are used to calculate the ideal threshold for 5.681 Å GNR in (20, 20) SWCNT, and the acquired thresholds of the first, second helix and whole system are 7, 4 and 13 respectively, in accordance with simulated values very well. So the two Formulas are proved applicable to estimate the quantity threshold for any GNR helical structure in SWCNT.

The obtained results shown in Fig. 3(d) demonstrate an interesting feature that the model of (7, 5) and (7, 4, 1) are changed into the structure of [6, 4, 2] with part of GNRs spontaneously translating into the inner spiral structure from the outermost layer, and the similar situations arise when wide GNRs filling SWCNT in Fig. 6 and Fig. 7, causing that the simulation threshold value is smaller than the predicted one which is calculated by Formulas 1. It can be suggested that this interesting configuration transformation is determined by the following three conditions:

i) Structure stability: New structures possess lower energy and better stability. The π-π stacking area between GNRs and SWCNT or the GNRs in different helices has been expanded in configuration transformation to reduce the total energy with the strengthened π-π stacking interaction, which is proved by the second changing stage of potential energy and π-π stacking area in Fig. 4(a), corresponding to the process of the outer GNRs into the inner helix as shown in Fig. 3(f);

ii) Initial arrangement: According to Fig. 3(a), 7 GNRs constitute the first spiral layer and no GNR enters into the inner helix spontaneously to reduce the energy of system, which exhibits that an inducing factor in the initial arrangement is needed to help GNRs break away from the original π-π stacking interaction between GNRs and SWCNT and overcome their energy barrier to achieve a more stable state. The inducing factor might be the opportune arrangement of GNR tails outside SWCNT leaded by their stochastic motion, and can also be the GNRs in inner layer if their quantities do not meet the theoretical maximum value;

iii) Demand of space: Enough space in SWCNT is imperative in order to achieve the successful configuration transformation for the composite system. Conversely, even if the system meets the above two conditions, the GNRs in the outer layer are still unable to enter into the inner layer when the space within the nanotubes is insufficient, as shown in Fig. 3(g).

In summary, this work proves that multiple GNRs can fill into cylindrical SWCNT to form the well-organized multiple helical configuration driven by the increasing π-π stacking area because system energy is released when carbon six-membered rings stacking. The final spiral configuration of GNRs in SWCNT is responsible for the combined effect of structure stability, initial arrangement and cavity space. So when the space is sufficient, the GNRs in outer helix can be inducted to enter into inner to improve the stability of whole system. For monolayer helix, the configuration forming time increases with its helical angle, illustrating that it will take longer time to form large helical angle configuration. The simulated threshold value of GNRs inside SWCNT increases by increasing SWCNT diameter or by decreasing GNR width, and the ideal quantity threshold can be estimated by empirical formula. Multilayered graphite has not been stripped in SWCNT, but formed a helical hybrid configuration which could be considered a metastable state due to the π-π stacking attractive interaction and enthalpically favorable T-shaped π interactions.

The above-mentioned discoveries are of great significance in predicting and controlling the GNR@SWCNT configuration, which might have some potential applications in contemporary nanomaterial engineering.

## Methods

Molecular dynamics method is used to study the configuration evolution of multiple GNRs filling the SWCNT. The force-field of condensed-phase optimized molecular potentials for atomistic simulation studies (COMPASS)^41^ which has been proven to be applicable in describing the mechanical properties of graphene sheets^42^ is applied to model the atomic interaction. The temperature is chosen as 298 K in the NVT canonical ensemble (number of particles, volume, temperature are constant). Andersen thermostat is employed to control the thermodynamic temperature and generate the correct statistical ensemble, while the kinetic temperature is kept constant by allowing the simulated system to exchange energy with a “heating bath”. The speed of atoms follows the Maxwell-Boltzmann distribution. The time integration of the Newton’s equation of motion is undertaken using the velocity Verlet algorithm. The simulation time step is 1.0 fs. Each system is simulated for sufficient time to reach equilibrium. Trajectory is recorded every 5.0 ps for further analysis.

## Additional Information

**How to cite this article**: Li, Y. *et al.* Multiple helical configuration and quantity threshold of graphene nanoribbons inside a single-walled carbon nanotube. *Sci. Rep.* **5**, 13741; doi: 10.1038/srep13741 (2015).

## References

[b1] Tsang S. C., Chen Y. K., Harris P. J. F. & Green M. L. H. A simple chemical method of opening and filling carbon nanotubes. Nature 372, 159–162 (1994).

[b2] Ajayan P. M., Stephan O., Redlich P. & Colliex C. Carbon nanotubes as removable templates for metal oxide nanocomposites and nanostructures. Nature 375, 564–567 (1995).

[b3] Nakanishi R., Kitaura R., Warner J. H., Yamamoto Y. & Arai S. Thin single-wall BN-nanotubes formed inside carbon nanotubes. Sci. Rep. 3, 1385 (2013). 23459405 10.1038/srep01385 PMC3587887

[b4] Choi W. Y., Kang J. W. & Hwang H. J. Structures of ultrathin copper nanowires encapsulated in carbon nanotubes. Phys. Rev. B 68, 380–383 (2003).

[b5] Gately R. D. & in het Panhuis M. Filling of carbon nanotubes and nanofibres. Beilstein J. Nanotech. 6, 508–516 (2015). 10.3762/bjnano.6.53 PMC4362020 25821693

[b6] Shao J., Yang C., Zhu X. & Lu X. Melting and freezing of Au nanoparticles confined in armchair single-walled carbon nanotubes. J. Phys. Chem. C 114, 2896–2902 (2010).

[b7] Hummer G., Rasaiah J. C. & Noworyta J. P. Water conduction through the hydrophobic channel of a carbon nanotube. Nature 414, 188–190 (2001). 11700553 10.1038/35102535

[b8] Govindaraj A., Satishkumar B. C., Nath M. & Rao C. N. R. Metal nanowires and intercalated metal layers in single-walled carbon nanotube bundles. Chem. Mater. 12, 202–205 (1999).

[b9] Sun F. W., Li H. & Liew K. M. Compressive mechanical properties of carbon nanotubes encapsulating helical copper nanowires. Carbon 48, 1586–1591 (2010).

[b10] Xu C. *et al.* 1D lanthanide halide crystals inserted into single-walled carbon nanotubes. Chem. Commun. 2427–2428 (2000).

[b11] Hirahara K. *et al.* Electron diffraction study of one-dimensional crystals of fullerenes. Phys. Rev. B 64, 115420 (2001).

[b12] Chen W. & Li H. How does carbon nanoring deform to spiral induced by carbon nanotube? Sci. Rep. 4, 7 (2014). 10.1038/srep03865 PMC3902510 24463737

[b13] Lv C. *et al.* Self-assembly of double helical nanostructures inside carbon nanotubes. Nanoscale 5, 4191–4199 (2013). 23334090 10.1039/c2nr33157h

[b14] Smith B. W., Luzzi D. E. & Achiba Y. Tumbling atoms and evidence for charge transfer in La2@C80@SWNT. Chem. Phys. Lett. 331, 137–142 (2000).

[b15] Won C. Y., Joseph S. & Aluru N. R. Effect of quantum partial charges on the structure and dynamics of water in single-walled carbon nanotubes. J. Chem. Phys. 125, 114701 (2006). 16999495 10.1063/1.2338305

[b16] Cicero G., Grossman J. C., Schwegler E., Gygi F. & Galli G. Water confined in nanotubes and between graphene sheets: a first principle study. J. Am. Chem. Soc. 130, 1871–1878 (2008). 18211065 10.1021/ja074418+

[b17] He Y., Sun G., Koga K. & Xu L. Electrostatic field-exposed water in nanotube at constant axial pressure. Sci. Rep. 4, 6596 (2014). 25318649 10.1038/srep06596 PMC4198863

[b18] Gogotsi Y., Libera J. A., Güvenç-Yazicioglu A. & Megaridis C. M. *In situ* multiphase fluid experiments in hydrothermal carbon nanotubes. Appl. Phys. Lett. 79, 1021–1023 (2001).

[b19] Koga K., Gao G. T., Tanaka H. & Zeng X. C. Formation of ordered ice nanotubes inside carbon nanotubes. Nature 412, 802–805 (2001). 11518961 10.1038/35090532

[b20] Gao H., Kong Y. & Cui D. Spontaneous insertion of DNA oligonucleotides into carbon nanotubes. Nano Lett. 3, 471–473 (2003).

[b21] Zou J., Liang W. & Zhang S. Coarse-grained molecular dynamics modeling of DNA-carbon nanotube complexes. Int. J. Numer. Meth. Eng. 83, 968–985 (2010).

[b22] Jiang Y. *et al.* Helical encapsulation of graphene nanoribbon into carbon nanotube. ACS Nano 5, 2126–2133 (2011). 21309562 10.1021/nn103317u

[b23] Li Y., Sun F. & Li H. Helical wrapping and insertion of graphene nanoribbon to single-walled carbon nanotube. J. Phys. Chem. C 115, 18459–18467 (2011).

[b24] Chuvilin A. *et al.* Self-assembly of a sulphur-terminated graphene nanoribbon within a single-walled carbon nanotube. Nat. Mater. 10, 687–692 (2011). 21822259 10.1038/nmat3082

[b25] Dutta S. & Pati S. K. Novel properties of graphene nanoribbons: a review. J. Mater. Chem. 20, 8207–8223 (2010).

[b26] Bai J. *et al.* Very large magnetoresistance in graphene nanoribbons. Nature Nanotech. 5, 655–659 (2010). 10.1038/nnano.2010.154 PMC2934897 20693988

[b27] Yang L., Cohen M. L. & Louie S. G. Excitonic effects in the optical spectra of graphene nanoribbons. Nano Lett. 7, 3112–3115 (2007). 17824720 10.1021/nl0716404

[b28] Ni Y., Yao K., Fu H., Gao G. & Zhu S. Spin seebeck effect and thermal colossal magnetoresistance in graphene nanoribbon heterojunction. Sci. Rep. 3, 1380 (2013). 23459307 10.1038/srep01380 PMC3587885

[b29] Furuhashi F. & Shintani K. Morphology of a graphene nanoribbon encapsulated in a carbon nanotube. AIP Adv. 3, 092103 (2013).

[b30] Yang X. *et al.* High-Efficiency loading and controlled release of doxorubicin hydrochloride on graphene oxide. J. Phys. Chem. C 112, 17554–17558 (2008).

[b31] Liu Z., Robinson J. T., Sun X. & Dai H. PEGylated nanographene oxide for delivery of water-insoluble cancer drugs. J. Am. Chem. Soc. 130, 10876–10877 (2008). 18661992 10.1021/ja803688x PMC2597374

[b32] Lebedeva I. V., Popov A. M., Knizhnik A. A., Khlobystov A. N. & Potapkin B. V. Chiral graphene nanoribbon inside a carbon nanotube: ab initio study. Nanoscale 4, 4522–4529 (2012). 22696165 10.1039/c2nr30144j

[b33] Talyzin A. V. *et al.* Synthesis of graphene nanoribbons encapsulated in single-walled carbon nanotubes. Nano Lett. 11, 4352–4356 (2011). 21875092 10.1021/nl2024678

[b34] Lim H. E. *et al.* Growth of carbon nanotubes via twisted graphene nanoribbons. Nat. Commun. 4, 2548 (2013). 24091379 10.1038/ncomms3548 PMC3806408

[b35] Główka M. L., Martynowski D. & Kozłowska K. Stacking of six-membered aromatic rings in crystals. J. Mol. Struct. 474, 81–89 (1999).

[b36] Wittung P., Nielsen P. E., Buchardt O., Egholm M. & Norde´n B. DNA-like double helix formed by peptide nucleic acid. Nature 368, 561–563 (1994). 8139692 10.1038/368561a0

[b37] Brodsky B. & Ramshaw J. A. M. The collagen triple-helix structure. Matrix Biol. 15, 545–554 (1997). 9138287 10.1016/s0945-053x(97)90030-5

[b38] Bets K. V. & Yakobson B. I. Spontaneous twist and intrinsic instabilities of pristine graphene nanoribbons. Nano Res. 2, 161–166 (2009).

[b39] Snir Y. & Kamien R. D. Entropically driven helix formation. Science 307, 1067 (2005). 15718461 10.1126/science.1106243

[b40] Janiak C. A critical account on π-π stacking in metal complexes with aromatic nitrogen-containing ligands. J. Chem. Soc., Dalton Trans., 3885–3896 (2000).

[b41] Sun H. COMPASS: An ab initio force-field optimized for condensed-phase applications overview with details on alkane and benzene compounds. J. Phys. Chem. B 102, 7338–7364 (1998).

[b42] Zheng Q., Geng Y., Wang S., Li Z. & Kim J.-K. Effects of functional groups on the mechanical and wrinkling properties of graphene sheets. Carbon 48, 4315–4322 (2010).

